# Exposure of Clinical MRSA Heterogeneous Strains to β-Lactams Redirects Metabolism to Optimize Energy Production through the TCA Cycle

**DOI:** 10.1371/journal.pone.0071025

**Published:** 2013-08-05

**Authors:** Mignon A. Keaton, Roberto R. Rosato, Konrad B. Plata, Christopher R. Singh, Adriana E. Rosato

**Affiliations:** 1 Department of Pathology and Genomic Medicine, Center for Molecular and Translational Human Infectious Diseases Research, The Methodist Hospital Research Institute, Houston, Texas, United States of America; 2 Metabolon, Inc., Durham, North Carolina, United States of America; National Institutes of Health, United States of America

## Abstract

Methicillin-resistant *Staphylococcus aureus* (MRSA) has emerged as one of the most important pathogens both in health care and community-onset infections. The prerequisite for methicillin resistance is *mecA*, which encodes a β-lactam-insensitive penicillin binding protein PBP2a. A characteristic of MRSA strains from hospital and community associated infections is their heterogeneous expression of resistance to β-lactam (HeR) in which only a small portion (≤0.1%) of the population expresses resistance to oxacillin (OXA) ≥10 µg/ml, while in other isolates, most of the population expresses resistance to a high level (homotypic resistance, HoR). The mechanism associated with heterogeneous expression requires both increase expression of *mecA* and a mutational event that involved the triggering of a β-lactam-mediated SOS response and related *lexA* and *recA* genes. In the present study we investigated the cellular physiology of HeR-MRSA strains during the process of β-lactam-mediated HeR/HoR selection at sub-inhibitory concentrations by using a combinatorial approach of microarray analyses and global biochemical profiling employing gas chromatography/mass spectrometry (GC/MS) and liquid chromatography/mass spectrometry (LC/MS) to investigate changes in metabolic pathways and the metabolome associated with β-lactam-mediated HeR/HoR selection in clinically relevant heterogeneous MRSA. We found unique features present in the oxacillin-selected SA13011-HoR derivative when compared to the corresponding SA13011-HeR parental strain that included significant increases in tricarboxyl citric acid (TCA) cycle intermediates and a concomitant decrease in fermentative pathways. Inactivation of the TCA cycle enzyme cis-aconitase gene in the SA13011-HeR strain abolished β-lactam-mediated HeR/HoR selection demonstrating the significance of altered TCA cycle activity during the HeR/HoR selection. These results provide evidence of both the metabolic cost and the adaptation that HeR-MRSA clinical strains undergo when exposed to β-lactam pressure, indicating that the energy production is redirected to supply the cell wall synthesis/metabolism, which in turn contributes to the survival response in the presence of β-lactam antibiotics.

## Introduction

*S. aureus* is a main pathogen responsible for a number of diseases ranging from skin and soft tissue infections to life-threatening endocarditis in hospitals and community settings [1]. The prerequisite for methicillin resistance located on SCC*mec* is *mecA*, which encodes a β-lactam-insensitive penicillin binding protein (PBP), PBP2a, that can continue to cross-link the cell wall once the native PBPs (i.e., PBP1−4) have been inactivated [2]. A characteristic of MRSA strains from hospital and community associated infections is their heterogeneous expression of resistance to β-lactam (heterotypic resistance [HeR]) [3]–[5] in which only a small portion (≤0.1%) of the population expresses resistance to oxacillin (OXA) ≥10 µg/ml, in contrast to other isolates in which most of the population expresses resistance to a high level (homotypic resistance [HoR]) [3]–[6]. In addition to *mecA*, the process of β-lactam-mediated HeR to HoR selection in MRSA strains is achieved by growing heterogeneous strains in the presence of sub-inhibitory concentrations of β-lactams [3]–[5]. As we demonstrated in previous studies, HeR MRSA strains clinically misinterpreted as MSSA (MICs to OXA: 2 µg/ml) were able to express a homogeneous high level of resistance (MICs: 256 µg/ml; HoR) when exposed to sub-inhibitory concentrations of OXA (0.5 µg/ml) [5]. Moreover, we have shown that β-lactam-mediated HeR/HoR selection was also associated with a mutational event that involved the triggering of a β-lactam-mediated SOS response and related *lexA* and *recA* genes [5]. Although the mechanism has been explored in detail, less is known about the cellular physiology of HeR-MRSA strains during the process of HeR/HoR selection by sub-inhibitory concentrations of β-lactams. Recent studies suggest that the basic physiology of *S. aureus* determines not only growth and survival but also pathogenicity and adaptation to stress conditions, including antibiotic pressure [7]. In this sense, it has been shown that *S. aureus* acquires resistance to vancomycin by adapting both its physiology and metabolism allowing its growth in the presence of the antibiotic [8]. In *S. aureus,* the tricarboxyl citric acid (TCA) cycle is essential for a majority of metabolic pathways 9–11. It serves as a central hub connecting catabolic energy gaining pathways with anabolic pathways like amino acid, fatty acid and nucleoside biosynthesis [11]. The central degradation product of glycolysis, pyruvate, is shunted into TCA cycle via acetyl-CoA [11]. Pyruvate dehydrogenase complex (PDC) catalyzes then the conversion of pyruvate to acetyl-CoA with a concomitant reduction of NAD+ to NADH and release of CO_2_ [12]. In the present study we used a combinatorial approach of microarray analyses with global biochemical profiling employing gas chromatography/mass spectrometry (GC/MS) and liquid chromatography/mass spectrometry (LC/MS) to investigate changes in metabolic pathways and the metabolome associated with β-lactam-mediated HeR/HoR selection in clinically relevant MRSA cells. By using these approaches, we found unique features present in SA13011 after β-lactam-mediated selection (HoR) that included significant increases in the TCA cycle intermediates citrate, cis-aconitate and fumarate and a significant decrease in lactate. Moreover, mechanistic studies based on the inactivation of *acnA*-*citB* (the first gene of the cycle), further demonstrated the functional significance of altered TCA cycle activity during the HeR/HoR selection. The present results reveal both the metabolic cost and the adaptation that HeR-MRSA clinical strains undergo when exposed to β-lactam pressure, indicating that the energy production is redirected to supply the cell wall synthesis/metabolism, contributing to the cell survival in the presence of β-lactam antibiotics. These studies involving the analyses of metabolic pathways in heterogeneous MRSA provide novel information which may represent an important contribution for future design of new targets against MRSA infections.

## Results

### Differential Gene Expression Analysis during OXA-mediated SA13011 HeR/HoR Selection Revealed Changes in Diverse Metabolic Pathways

In an attempt to determine differentially expressed genes associated with β-lactam-mediated HeR/HoR selection, we performed gene expression analysis using spotted DNA microarrays as previously described [13]. Pair-wise comparisons were made in biological triplicates between SA13011- HeR and SA13011-HoR isogenic strains. SA13011-HeR (OXA MIC: 2 µg/ml) was grown in absence and presence of sub-inhibitory concentrations of OXA (0.5 µg/ml) leading to SA13011-HoR (Oxacillin MIC: ≥256 µg/ml) and collected at similar exponential growth phase as described both in Methods and previously [5], [13]. The extension of microarrays analyses reported here focused specifically in genes associated with metabolic pathways. These results are based on a series of statistical analysis (filtering) where ratios of Cy3 and Cy5 signals were converted to log2 values and cutoff was set at above 1 (present) or below -1 (absent), as previously described [13], [14]. The ORFs considered here as differentially expressed are those which log2 ratios of Cy3/Cy5 signals equals 1 (two-fold change) in at least three out of four independent experiments [13], [14].

A total of 230 genes were found differentially expressed, evenly distributed between up- and down-regulated genes (Table 1); these genes were classified in six distinct groups. The most represented group of differentially expressed genes belongs to the functional category carbohydrate transport and metabolism (22%) followed by genes involved in amino acid transport and metabolism (16%), energy production and conversion (10%) and cell wall, membrane, envelope biogenesis (5%; Table 1).

**Table 1:** Differential gene expression analysis of metabolism-related pathways between strains SA13011-HoR *vs*. SA13011-HeR.

ORF	Gene	Fold change	Name	Description	
**Carbohydrate transport and metabolism**					
**SA0258**	*rbsK*	3.7	ribokinase	Catalyses the phosphorylation of ribose to ribose-5-P	
**SA0259**	*rbsD*	5.3	D-ribose pyranase	Catalyzes the conversion between beta-pyran and beta-furan forms of D-ribose.	
**SA0260**	*rbsU*	4.2	hypothetical protein	Putative ribose uptake protein rbsU.	
**SA0510**	*araB*	3.5	Ribulokinase	Catalyzes the phosphorylation of ribulose to ribulose-5-P	
**SA1140**	*glpF*	4.8	glycerol uptake facilitator	Facilitates diffusion of glycerol into the cells.	
**SA0433**		−23.1	alpha-glucosidase	Converts trehalose-6-P into D-glucose 6-P.	
**SAS0431**		−11.2	sugar-specific PTS transport system, IIBC component	Phosphotransferase transport system (PTS),	
**SAS0432**		−19.8	putative glycosyl hydrolase	Converts trehalose-6-P into glucose-6-P.	
**SAR2247**	*mtlD*	5.2	mannitol-1-phosphate 5-dehydrogenase	Catalyzes: D-mannitol-1-P+NAD+ = D-fructose-6-P+NADH	
**SA1336**		2	glucose-6-phosphate 1-dehydrogenase	Catalyzes: D-glucose-6-P+NADP+ = D-glucono-1,5-lactone-6-P+NADPH	
**SA1065**	*cfxE*	2.3	hypothetical protein	pentose phosphate pathway	
**SACOL1124**	*lctP*	**−**2.4	hypothetical protein	Transports L-lactate across the membrane.	
**SA0106**		**−**20.3	hypothetical protein	homolog of L-lactate permease lctP	
**SA2156**		**−**4.6	hypothetical protein	Maltose/maltodextrin transport permease	
**SAS0164**		2.9	glucose-specific PTS transporter protein, IIABC component	Phosphotransferase transport system (PTS), glucose-specific.	
**SA0099**		−5.5	putative PTS transport system, IIABC component	Putative phosphotransferase transport system (PTS), mannose specific.	
**SAS2527**		8.9	hypothetical protein	Phosphotransferase transport system (PTS)	
**SA2320**	*glcA*	−4.6	PTS system glucose-specific EIICBA component	Phosphotransferase transport system (PTS, glucose-specific).	
**SA2434**		4.2	PTS system EIIBC component	Putative phosphotransferase transport system (PTS), EIIBC component.	
**SA0186**	*ptsG*	−4	PTS system glucoside-specific IICBA component	Phosphotransferase transport system (PTS), glucoside specific.	
**SA0325**	*glpT*	−2.7	glycerol-3-phosphate transporter	Transport of glycerol-3-P.	
**SA1533**	*ackA*	−3.1	acetate/propionate kinase	Involved in pyruvate, propanoate, taurine and hypotaurine metabolism (conversion of acetate to acetyl-P and propanoate into propanoyl-P)	
**SA1236**	*acyP*	3.1	acylphospha-tase	Involved in pyruvate metabolism, glycolysis/gluconeogenesis.	
**SA1554**	*acsA*	4	acetyl-coenzyme A synthetase	Conversion of acetate and CoA to acetyl-CoA.	
**SA1609**	*pckA*	5.6	Phosphoenol-pyruvate carboxykinase	Involved in the TCA cycle and pyruvate metabolism (catalyzesATP+oxaloacetate = ADP+phosphoenolpyruvate+CO_2_)	
**SA1184**	*citB*	6.3	aconitate hydratase	Involved in the TCA cycle (conversion of citrate to isocitrate).	
**SA1244**	*odhB*	2.2	Dihydrolipo-amide acetyltrans-ferase	Involved in the TCA cycle and lysine degradation.	
**SA1518**	*citZ*	8.9	citrate synthase	Catalyzes the first step in the TCA cycle.	
**SAS1622**	*citC*	11	isocitrate dehydro-genase	Involved in the TCA cycle (converts isocitrate to alpha ketoglutarate).	
**SAR1942**	*citG*	2.5	fumarate hydratase, class-II	Involved in the TCA cycle (converts (S)-malate to fumarate and water).	
**SA1089**	*sucD*	3.3	succinyl-CoA synthetase alpha subunit	Catalyzes the only substrate-level phosphorylation in the TCA cycle.	
**SA0963**	*pycA*	−3	pyruvate carboxylase	Involved in the TCA cycle, alanine and aspartate metabolism, pyruvate metabolism.	
**SA1510**	*gapB*	4.8	glyceraldehyde 3-phosphate dehydrogenase	Involved in glycolysis and glyconeogenesis.	
**SA1845**		−2.2	hypothetical protein similar to fructokinase	Catalyzes conversion of fructose to fructose-6-P	
**Energy metabolism**					
**SA1927**	*fbaA*	−2.3	fructose-bisphosphate aldolase	Involved in gluconeogenesis	
**SA2204**	*gpmA*	−4	2,3-bisphosphoglycerate-dependent phosphoglycerate mutase	Involved in glycolysis/gluconeogenesis	
**SAS2401**		2.9	hypothetical protein	Putative fructose-1,6-bisphosphatase III involved in glycolysis/gluconeogenesis, pentose phosphate pathway and fructose and mannose metabolism	
**SA0212**		−3.1	hypothetical protein	Similar to sugar phosphate isomerases/epimerases	
**SA2102**		−4	hypothetical protein	Putative formate dehydrogenase	
**SA0367**	*nrfA*	2.4	NADPH-dependent oxidoreductase	Involved in maintenance of the cellular redox state and the disulfide stress response	
**SA2312**	*ddh*	−10.8	D-lactate dehydroge-nase	Catalyzes the formation of pyruvate from lactate	
**SA0218**	*pflB*	−10.5	formate acetyltrans-ferase	Catalyzes a key step in anaerobic glycolysis (conversion of pyruvate and CoA to formateacetyl-CoA)	
**SA0232**	*lctE*	−5.7	L-lactate dehydro-genase	Catalyzes conversion of pyruvate (the final product of glycolysis) to lactate in the absence of oxygen	
**Oxidative phosphorylation**					
**SA1241**	*qoxD*	−4.8	probable quinol oxidase subunit 4	Involved in oxidative phosphorylation pathway.	
**SA0910**	*ppaC*	−2.7	putative manganese-dependent inorganic pyrophosphatase	Involved in oxidative phosphorylation pathway, catalyzes the hydrolysis of pyrophosphate to phosphate.	
**SA1735**	*ctaA*	−3.5	cytochrome oxidase assembly protein	Cytochrome oxidase assembly protein.	
**SA0684**		−4.8	hypothetical protein	Similar to transmembrane efflux pump protein.	
**Aminoacid transport and metabolism**					
**SA1531**	*Ald*	3.3	alanine dehydrogenase	Role in cell wall synthesis, as L-alanine is an important constituent of the peptidoglycan layer.	
**SA1365**	*gcvPB*	2.9	glycine dehydrogenase subunit 2	Catalyzes the degradation of glycine.	
**SA1366**	*gcvPA*	4.8	glycine dehydrogenase subunit 1	Glycine cleavage system P-protein subunit 1.	
**SA1367**	*gcvT*	5.3	aminomethyltransferase	Glycine cleavage system aminomethyltransferase T.	
**SA2226**		7.2	hypothetical protein	Similar to D-serine/D-alanine/glycine transporter.	
**SA2327**		−5.9	pyruvate oxidase	Similar to pyruvate oxidase (catalyzes formation of acetyl phosphate from pyruvate).	
**SA2318**		8.4	hypothetical protein	Similar to L-serine dehydratase (catalyses deamination of serine to form pyruvate).	
**SA0818**	*rocD*	4.2	ornithine-oxo-acid transaminase	Involved in urea cycle and metabolism of amino groups.	
**SA2341**	*rocA*	4.8	1-pyrroline-5-carboxylate dehydrogenase	Involved in L-proline degradation into L-glutamate	
**SA1585**		3	hypothetical protein	Similar to proline dehydrogenase.	
**SA1436**		8.2	hypothetical protein	Similar to allophanate hydrolase subunit 2	
**SA1707**		3	hypothetical protein	Predicted glutamine amidotransferase	
**SA0717**		2	hypothetical protein	acetyltransferase (isoleucine patch superfamily)	
**SA2229**		−2.2	hypothetical protein	Similar to amino acid transporters	
**SA0180**		−2.3	hypothetical protein	Similar to branched-chain amino acid transport system carrier protein.	
**SA2254**	*opp-1B*	−2.9	oligopeptide transporter putative membrane permease domain	dipeptide/oligopeptide/nickel transport systems.	
**SA2200**		−2.4	hypothetical protein	Similar to ABC transporter	
**SA2227**		7.6	hypothetical protein	Similar to gamma-aminobutyrate permease and related permeases.	
**SA1169**		−2.3	gamma-aminobutyrate permease	Amino acid transporter.	
**SA1718**	*putP*	−2.6	high affinity proline permease	Proline permease	
**SA0859**		3	hypothetical protein	Similar to oligoendopeptidase F.	
**Cell wall associated genes**					
**SA1343**		2.3	hypothetical protein	Similar to tripeptidase	
**SA1283**	*pbp2*	2.6	penicillin binding protein 2	Membrane carboxypeptidase	
**SA0038**	*mecA*	2.8	penicillin binding protein 2A	Membrane transpeptidase	
**SA1206**	*femA*	3.0	factor essential for expression of methicillin resistance	Factor essential for expression of methicillin resistance; involved in the formation of the staphylococcal pentaglycine interpeptide bridge	
**SA0244**	*tagF*	5.0	putative glycosyl/glycerophos-phate transferase	Similar to teichoic acid biosynthesis protein F (TagF)	
**SAR2242**	*glmS*	5.3	D-fructose-6-phosphate amidotransferase	Catalyzes the first step in hexosamine metabolism (converts fructose-6P into glucosamine-6P)	
**SA1475**		3.0	putative cell shape determinant mreC	Rod shape-determining protein	
**SAS0648**	*uppP*	3.0	undecaprenyl pyrophosphate phosphatase	Participates in peptidoglycan biosynthesis; involved in bacitracin resistance	
**SA2437**		2.8	N-acetylmuramoyl-L-alanine amidase precursor	Autolysin precursor	
**SA0185**	*murQ*	7.9	N-acetylmuramic acid 6-phosphate etherase	Involved in N-acetylmuramic acid degradation	
**SA1183**	*opuD*	−2.7	glycine betaine transporter	Transporter	
**SA1987**		−6.4	hypothetical protein	Probable glycine betaine transporter opuD homolog.	
**SA0659**		−3.6	hypothetical protein	Similar to CsbB stress response protein.	
**SA0511**		−2.2	hypothetical protein	Similar to nucleoside-diphosphate-sugar epimerases.	
**SA1141**	*glpK*	7.2	glycerol kinase	Involved in the regulation of glycerol uptake and metabolism, glycerolipid metabolism (catalyzes glycerol to glycerol 3-P).	
**SA0432**	*treP*	−13.4	PTS enzyme II, phosphoenol-pyruvate-dependent, trehalose-specific	Phosphotransferase transport system (PTS), trehalose-specific.	

### Carbohydrate Transport and Metabolism

Differentially regulated genes in the group of carbohydrate transport and metabolism included genes involved in the utilization of ribose as a carbon source, namely *rbsK, rbsD*, and *rbsU*, (SA0258, SA0259 and SA0260, respectively) all of which were up-regulated during HoR selection. RbsD functions as an ABC-type ribose transporter which also catalyzes conversion between β-pyran and β-furan forms of D-ribose [15]; *rbsU* encodes for a hypothetical ribose uptake protein and *rbsK* encodes for ribokinase that catalyzes the phosphorylation of ribose to ribose-5-phosphate, the initial step in ribose metabolism. Ribose-5-phosphate serves as the substrate in pentose phosphate pathway for energy production as well as the carbon source in tryptophan, histidine and nucleotide synthesis [15]. Elevated expression of *rbsK*, *rbsD*, and *rbsU* may suggest intensified use of D-ribose as energy and carbon source by SA13011-HeR during the selection process.

In the same group we observed the phosphoenolpyruvate-dependent sugar phosphotransferase system (PTS), a major form of carbohydrate transport involved in the translocation across cell membrane and phosphorylation of incoming carbohydrates [16]. A pronounced reduction in the expression of trehalose specific, phosphoenolpyruvate-dependent phosphotransferase system (13-fold; *treP*, SA0432) and α-glucosidase (23-fold; SA0433) that converts trehalose into D-glucose-6-phosphate was observed. Moreover, expression of several additional sugar phosphotranspherase transport systems were down-regulated during HeR/HoR selection including putative mannose specific PTS (SA2527), glucose specific PTS (*glcA*, SA0183), putative fructose specific PTS (SA2434), glucoside specific PTS (*ptsG*, SA2326) and glucose specific PTS (SA1566). A group of hypothetical proteins predicted to be related to carbohydrate metabolism were downregulated during HeR/HoR selection, including lactate permease (*lctP*, SA0106), probable homolog of lactate permease (SA2156), hypothetical maltose/maltodextrin permease homolog (SA0209) and glycerol-3-phosphate transporter (*glpT*, SA0325).

### Amino Acid Transport and Metabolism and Cell Wall Precursors

The second group of genes displaying differential expression between SA13011-HeR/−HoR strains included up-regulation of genes encoding for enzymes involved in degradation of peptidoglycan constituents. Alanine is an important component of *S. aureus* cell wall, where it represents three out of five amino acids in the stem peptides of peptidogylcan [17]. Alanine dehydrogenase (*ald*, SA1531), which hydrolyses L-alanine to ammonia, pyruvate and NADH, were up-regulated 3.3 fold during selection. Three genes involved in glycine degradation, the constituent of pentaglycine bridges of peptidoglycan [18], were also found to be up-regulated. Glycine dehydrogenase subunit 2 (*gcvPB*, SA1365), subunit 1 (*gcvPA*, SA1366) and aminotransferase (*gcvT*, SA1367) are co-expressed and form an operon as judged by their close location, direction of expression, and existence of the *gcv* operon in *E. coli*. GcvPB, GcvPA and GcvT constitute the glycine cleavage system involved in glycine degradation, which cleavages glycine into CO_2_ and NH_3_, generating NADH and one carbon unit [19]. Elevated expression of *ald* and genes that encode for enzymes of glycine cleavage system may suggest an intensified catabolism of two major components of peptidoglycan, glycine and alanine, and implies intense cell wall turn-over during SA13011-HoR selection. Another gene up-regulated and involved in amino acid catabolism encodes for L-serine dehydratase (SA2318). This enzyme catalyzes the conversion of L-serine to pyruvate and NH_3_. Up-regulation of SA2318 suggests intensified serine degradation and generation of pyruvate which will then serve as the substrate in multiple metabolic pathways. Its elevated expression also coincides with up-regulation of the hypothetical transporter of serine/alanine/glycine (SA2226).

Catabolism of proline also seems to be intensified during HeR/HoR selection since the gene encoding for a protein similar to proline dehydrogenase (SA1585) was up-regulated, as was the gene encoding the enzyme 1-pyrroline-5-carboxylate dehydrogenase (*rocA*, SSA2341) that catalyzes the second step in proline degradation. Several other genes encoding for proteins involved in the urea cycle were found to be up-regulated, including allophanate hydrolase subunit 2 (SA1436) and glutamine amidotransferase (SA1707).

### Energy Production

A number of genes involved in glycolysis, acetate metabolism, and the TCA cycle were found to be differentially expressed during HeR/HoR selection. Multiple genes involved in glycolysis/gluconeogenesis were found to be down-regulated, including fructose-biphosphate aldolase (*fba*, SA1927) and 2,3-biphosphoglycerate-dependent phosphoglycerate mutase (*gpmA*, SA2204). Fba catalyzes the conversion of fructose-1,6-bisphosphate into dihydroxyacetone phosphate while glyceraldehyde-3-phosphate (GpmA) is responsible for converting glycerate-3-phosphate into glycerate-2-phosphate. We also observed a marked down-regulation of L-lactate dehydrogenase (*lctE*, SA0232) and D-lactate dehydrogenase (*ddh*, SA2312), which interconvert pyruvate and lactate. Genes encoding enzymes involved in gluconeogenesis were similarly down-regulated including pyruvate carboxylase (*pycA*, SA0963), which catalyzes the conversion of pyruvate into oxaloacetate, and fructose-1,6-bisphosphatase III (SA2401). The only glycolytic gene observed to be up-regulated during the HeR/HoR selection was glyceraldehyde-3-phosphate dehydrogenase (*gapB*, SA1510).

In contrast to glycolytic genes, the expression of many TCA cycle genes was found to be up-regulated. These include citrate synthase (*citZ*, SA1518), aconitate hydratase (*citB*, SA11884), isocitrate dehydrogenase (*citC,* SAS1622), and a subunit of α-ketoglutarate dehydrogenase (*odhB*, SA1244). Another gene related to the TCA cycle also found to be up-regulated was *acsA* (SA1554); this gene codes for the enzyme responsible of converting acetate into acetyl-CoA, which can be then be used as carbon or energy source [20]. Elevated expression of *acsA* coincided with elevated expression of aldehyde dehydrogenase homologue (*aldA*, SA0162) and acylphosphatase (*acyP,* SA1236), both involved in acetate generation. AldA catalyzes the conversion of acetaldehyde into acetate with simultaneous generation of NADPH while AcyP generates acetate and ATP from acetyl-phosphate. Elevated expression of these genes, which generate acetate and energy via ATP and NADPH, and the concomitant reduction in expression of acetate/propionate kinase (*ackA*, SA1533), which stores energy in the form of acetyl phosphate, suggest that acetate generation may constitute one of the energy sources that feeds into the TCA cycle during outgrowth of SA13011-HoR. Interestingly, we identified several down-regulated genes encoding for enzymes involved in oxidative phosphorylation including quinolone oxidase subunit 4 (*qoxD*, SA0910), manganese-dependent inorganic pyrophosphatase (*ppaC*, SA1735), and cytochrome assembly protein (*ctaA*, SACOL1124). These changes suggest limited aerobic metabolism and limited energy production by oxidative phosphorylation during outgrowth of SA13011-HoR.

### Cell Wall Synthesis

Other genes whose expression was also found elevated during HeR/HoR selection were *glpF* and *glpK* (SA1140 and SA1141, respectively). These genes encode for glycerol uptake facilitator and for glycerol kinase, respectively [21]. Glycerol-3-phoshate is substrate for lipid biosynthesis including lipoteichoic and teichoic acids, which are important components of both cell membrane and cell wall. Increased expression of *glpF* (approximately 5-fold) and *glpK* (above 7-fold) during HeR/HoR selection may suggest an intensified transport of glycerol and synthesis of glycerol-3-phosphate during outgrowth of SA13011-HoR. Along with PBP2a (*mecA*) up-regulation, expression of *pbp2* (PBP2; SA1283) was also found to be elevated, consistent with the requirement of their cooperative effect (transglycosylase domain of PBP2 and the transpeptidase activity of PBP2a) for methicillin resistance in *S. aureus* [22]. Another important gene found to be up-regulated in HoR was *femA* (SA1206), a factor essential for methicillin resistance. FemA is responsible for incorporation of glycines 2 and 3 into pentaglycine cross-bridges that allows high crosslinking of peptidoglycan, a hallmark of the *S. aureus* cell wall [23]. An additional interesting gene found to be up-regulated was (SA0244), a putative glycosyl/glycerphosphate transferase that participates in teichoic and lipoteichic acids biosynthesis; SA0244 exhibits homology with TagF which adds glycerol-phosphate units to the growing chains of poly-glycerols bound to *N*-acetylglucosamine-β-(1–4)-*N*-acetylmannosamine and linked to lipid carrier, undecaprenyl-pyrophospate [24]. It has been suggested that the large multienzyme complex of which TagF is a part localizes at sites of cytoplasmatic membrane determined by the localization of MreC [24]. These observations may suggest that MreC participates not only in teichioc/lipteichoic acids biosynthesis, but also in peptidoglycan biosynthesis by directing the localization of enzymatic biosynthetic machineries to proper sites of synthesis. Proof of the intensity and correlation of these processes may come from up-regulation of undecaprenyl pyrophosphate phosphatase (*uppP*, SA0648). Undecaprenyl phosphate (bactoprenol) is the carrier lipid on which intermediates of peptidoglycan, teichoic/lipoteichoic acids and other components of cell wall are assembled and transported across cytoplasmic membrane. Elevated expression of *uppP* suggests intensified recycling of the lipid carrier which is necessary during intense cell wall synthesis. Concurrent elevation of gene expression involved in peptidoglycan synthesis (*pbp2, pbp2a*, *femA*), teichoic/lipoteichic acids polymerase (*tagF*) and *mreC*, suggests organized, intensified and perhaps coordinated synthesis of murein, teichoic and lipoteichoic acids during outgrowth of the HoR derivative.

**Figure 1:**
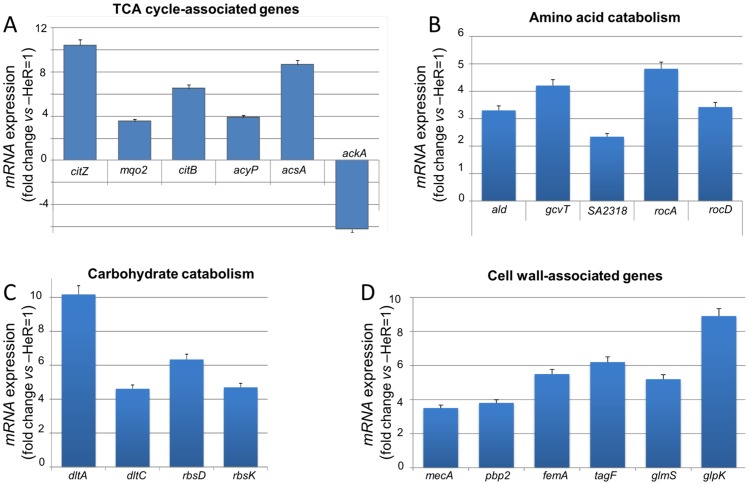
Quantitation of mRNA levels of TCA cycle-, amino-acid catabolism-, carbohydrate catabolism- and cell wall-associated genes by real-time RT-PCR. RNA was prepared from SA13011-HeR and its highly resistant derivative SA13011-HoR (SA13011+ OXA 0.5 µg/ml) cells, collected at exponential phase of growth, as described in Materials and Methods. Relative fold change values of specific mRNAs in SA13011-HoR vs. SA13011-HeR (reference value = 1) are shown on the vertical axis. Relative fold change values representing the means of at least three biological replicates of specific mRNAs ± standard error of the mean (SEM), sampled in triplicate to minimize error by inter- and intra-samples, are shown on the vertical axis; 16S rRNA was used as an internal control. Differences between the mean values were analyzed using a one-way analysis of variance (ANOVA). A *P* value of <0.01 was considered statistically significant. Oligonucleotide primers are shown in Table S2.

Validation of the metabolic changes in gene expression regulated during HeR/HoR selection identified by microarray analysis was performed by Real-Time RT-PCR by using RNAs collected from SA13011-HeR and -HoR cells. Consistent with the microarray analysis, we observed a 10-fold increase in *citZ* and a 6-fold increase in *citB* expression (Figure 1A). We also measured expression of *mqo2* which encodes malate dehydrogenase and found its expression was also elevated in SA13011-HoR. We confirmed changes reporting on acetate metabolism (*acyP, acsA,* and *ackA;* Figure 1A), proline and ornithine metabolism [*rocA*, (1-pyroline-5-carboxylate dehydrogenase); *rocD* (ornithine-oxo-acid transaminase; Figure 1B], and ribose metabolism (*rbsD;* Figure 1C) that were observed in the microarray analysis (Table 1). Additionally, we determined increased expression of *dltA* [D-alanine-poly (phosphoribitol) ligase subunit 1] and *dltC* [D-alanine-poly (phosphoribitol) ligase subunit 2] (Figure 1C), both genes encoding for enzymes involved in ribitol metabolism [25]. Expression analysis of cell wall associated genes included genes related to the expression of methicillin resistance including PBP2a (*mecA*, 6-fold increase) and PBP2 (*pbp2*, 4-fold increase), as well as genes associated with peptidoglycan cross-linking (*femA*; 5.8-fold increase) (Figure 1D). Consistent with their role, expression of glucosamine-6-phosphate synthase (*glmS*), important for production of a major building block of peptidoglycan, was also found up-regulated in SA13011-HoR strain (Figure 1D). Similar results were obtained during HeR/HoR selection of the heterogeneous MRSA strain SA43002 (phenotypically similar to SA13011) (Fig. S3).

#### Global biochemical profiling during β-lactam-mediated HeR/HoR selection

**Figure 2:**
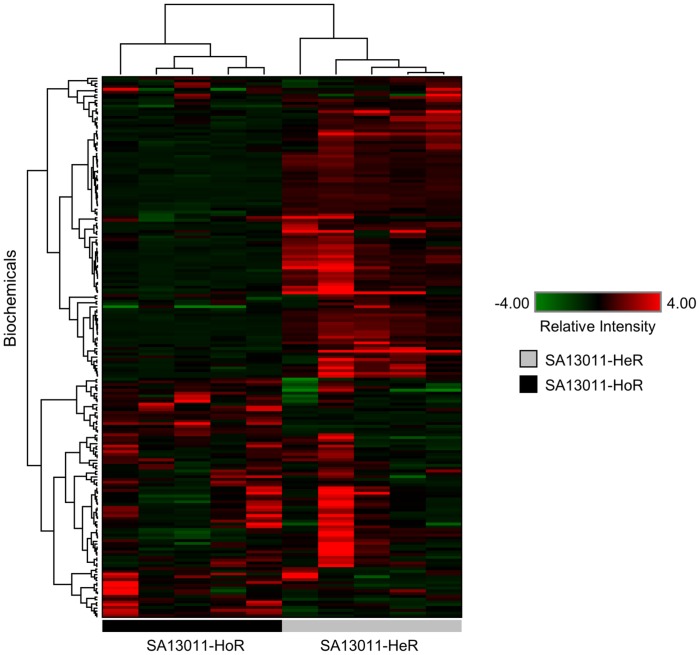
Heat map of metabolite levels profiled in the heterogeneous SA13011 strain (HeR) and its highly homogeneous methicillin resistant derivative SA13011-HoR (SA13011+ OXA 0.5 µg/ml). Red indicates high levels and green indicates low levels of each biochemical arranged on they-axis.

In order to identify and characterize metabolic changes associated with β-lactam-mediated HeR/HoR selection, untargeted, global biochemical profiling was performed for SA13011 during β-lactam-mediated HeR/HoR selection, as described in Methods. Cells were all collected at similar phase of growth (OD_600_ 0.7). A total of 194 biochemicals were identified and categorized into amino acid, carbohydrate, fatty acid, nucleotide, and cofactor classes. From these 194 metabolites, 98 biochemicals were significantly altered in their levels when SA13011-HoR was compared to SA13011-HeR (Table 2). Hierarchical cluster analysis of the resulting biochemical profiles revealed the existence of profound differences associated with β-lactam heterogeneous resistance (Figure 2). In fact, the SA13011-HoR profile was vastly different from SA13011-HeR, clearly emphasizing the metabolic adaptation that the strain undergoes when under β-lactam pressure. Importantly, many of the changes observed in the global biochemical profiles are consistent with the differential gene expression observed between the SA13011-HeR and SA13011-HoR strains described above.

**Table 2:** Summary showing the number of biochemicals statistically significantly different (p<0.05) between SA13011-HoR vs. SA13011-HeR.

Welch's Two Sample t-Tests	Number of biochemicals with p≤0.05	Number of biochemicals increased p≤0.05	Number of biochemicals decreased p≤0.05
**SA13011-HoR vs. SA13011-HeR**	98	15	83

### Energy Metabolism and Resistance to β-lactam

**Figure 3:**
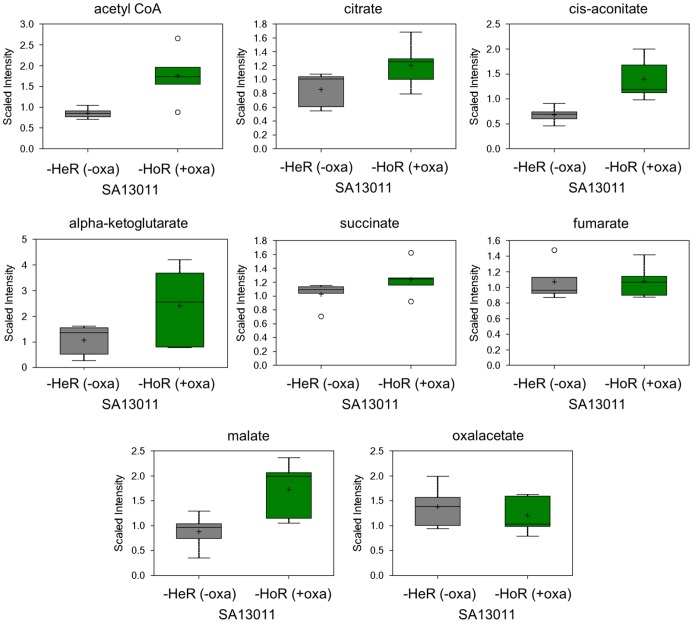
Analysis of biochemicals associated with the TCA cycle determined by global biochemical profiling across SA13011-HeR and SA13011-HoR (SA13011+ OXA 0.5 µg/ml) derivative.

**Figure 4:**
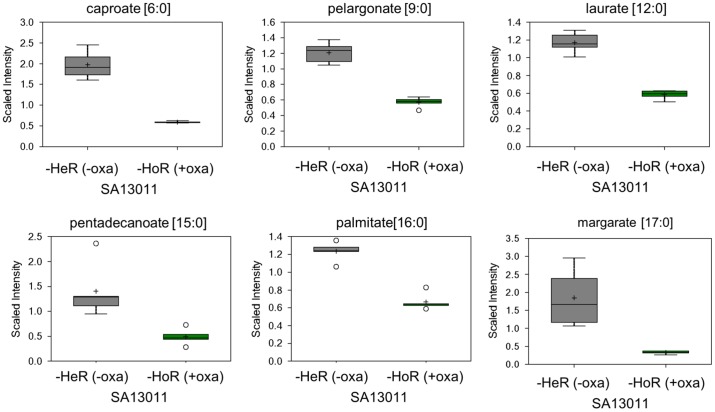
Analysis of biochemicals associated with sources of acetyl-CoA generation from β-oxidation of fatty acids in SA13011-HeR and SA13011-HoR (SA13011+ OXA 0.5 µg/ml) during β-lactam mediated HeR/HoR selection.

**Figure 5:**
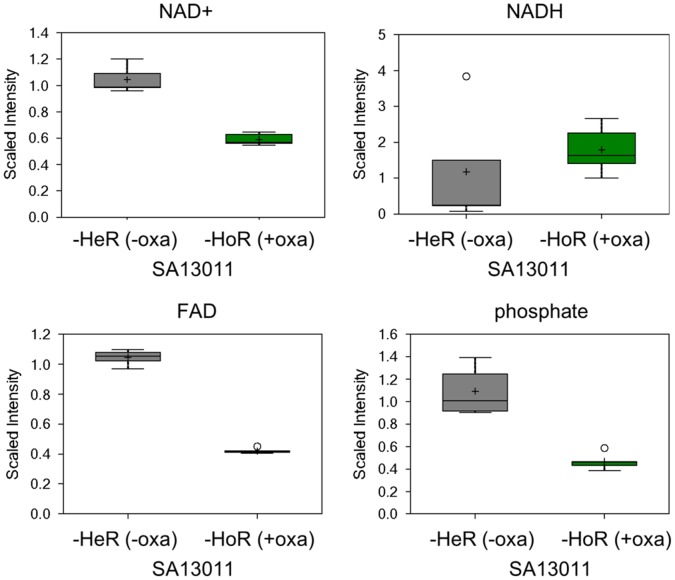
Analysis of biochemicals corresponding to energy metabolism components NADH, NAD+, FAD, phosphate and pyrophosphate (PPi) levels during β-lactam mediated SA13011-HeR/HoR selection.

Consistent with the upregulation of several TCA cycle genes during HoR selection, many of the TCA intermediates were elevated in the SA13011-HoR strain as compared to SA13011-HeR, with significant increases in the levels of cis-aconitate and malate (Figure 3; Table S1). These changes suggest that the SA13011-HoR strain is potentially capable of higher energy production and increased biosynthetic capabilities than the parental SA13011-HeR strain that may allow the HoR-derivative strain to respond to inhibition of wall synthesis by β-lactams. SA13011-HoR also displayed significantly higher levels of acetyl-CoA, which can be generated from glycolysis, β-oxidation of fatty acids, or acetate. Glycolysis did not appear to increase during the selection process, as 3-phosphoglycerate and phophoenolpyruvate were unchanged (Table S1). Instead, the majority of medium chain and long chain free fatty acids were found to be significantly decreased in SA13011-HoR, indicating increased utilization of this energy source (Figure 4). Consistent with an increase in fatty acid oxidation and TCA activity, acetyl-CoA (Figure 3) and NADH (Figure 5) levels rose after OXA-mediated HeR/HoR selection (SA13011-HoR). This was accompanied by a decrease in NAD levels (Figure 5) suggesting that the increased NADH was not being utilized. Together with diminished phosphate levels (Figure 5) and the decreased expression of a number of genes involved in oxidative phosphorylation in SA13011-HoR cells, including a subunit of FoF1-ATP synthase (*atpC*) and a protein required for cytochrome oxidase assembly (*ctaA*; Table 1), these changes indicate a decrease in oxidative phosphorylation during the selection process.

### Amino Acid Catabolism

Additional biochemical changes associated with SA13011-HoR included reduction in several amino acids and their metabolites (Fig. S1; Table S1). These changes may reflect altered utilization, synthesis, and catabolism. The latter possibility is supported by increased expression of genes involved in amino acid degradation. For example, a dramatic reduction in glycine levels was observed in SA13011-HoR cells (Fig. S1) coincidently with upregulation of genes that comprise the glycine cleavage system (Table 1). Similarly, levels of alanine, serine, proline, and ornithine were reduced in HoR cells, consistent with the increased expression of *SA2318* and *ald*, *SA1585* and *rocA*, and *rocD,* respectively. Catabolism of alanine and serine produce pyruvate that can then be converted to acetyl-CoA, while catabolism of proline and ornithine produce glutamate which can be converted to α-ketoglutarate, and thus contribute to the TCA cycle. Although most amino acids and their metabolites were decreased in during HoR selection, glutamine, which is synthesized from glutamate and is a component of peptidoglycan, was dramatically increased. Finally, the branched chain amino acid metabolites α-hydroxyisocaproate, α-hydroxyisovalerate, and 2-hydroxy-3-methylvaleratere were increased in HoR cells (Table S1), indicating catabolism of leucine, isoleucine, and valine which ultimately produces acetyl-CoA and succinyl-CoA that enter the TCA cycle.

### Markers of Membrane and Cell Wall Remodeling

While most fatty acids decreased compared to the HoR population (Table S1), several branched-chain fatty acids and the saturated 20 carbon fatty acid arachidate were significantly increased in OXA-selected samples (SA13011-HoR) suggesting they may play a specific role in antibiotic resistance. Since increases in a free fatty acid can arise from increased synthesis, remodeling of the cell membrane, or deconjugation from cell wall components, increases in 15-metylpalmitate/2-methylpalmitate and arachidate may reflect membrane and cell wall remodeling mediated by OXA treatment.

### Oxidative DNA Damage

Another difference between SA13011-HeR/HoR strains corresponded to a marked decreased in the levels of glutathione (Fig. S2), which may be associated in these cells with both increased oxidative stress and activation of a DNA damage response, as we previously reported during SA13011-HeR/HoR selection [5]. It is plausible that the loss of this key antioxidant compound may contribute to increased mutation rates and the selection of the homotypic resistant phenotype [5]. Thus, decreased glutathione may predispose cells to enhancing β-lactam resistance mechanisms by increasing oxidative DNA damage and consequently the SOS response that is required for OXA-mediated HeR/HoR selection [5].

#### TCA cycle is functionally associated with-β-lactam-mediated SA13011 HeR/HoR selection

**Figure 6:**
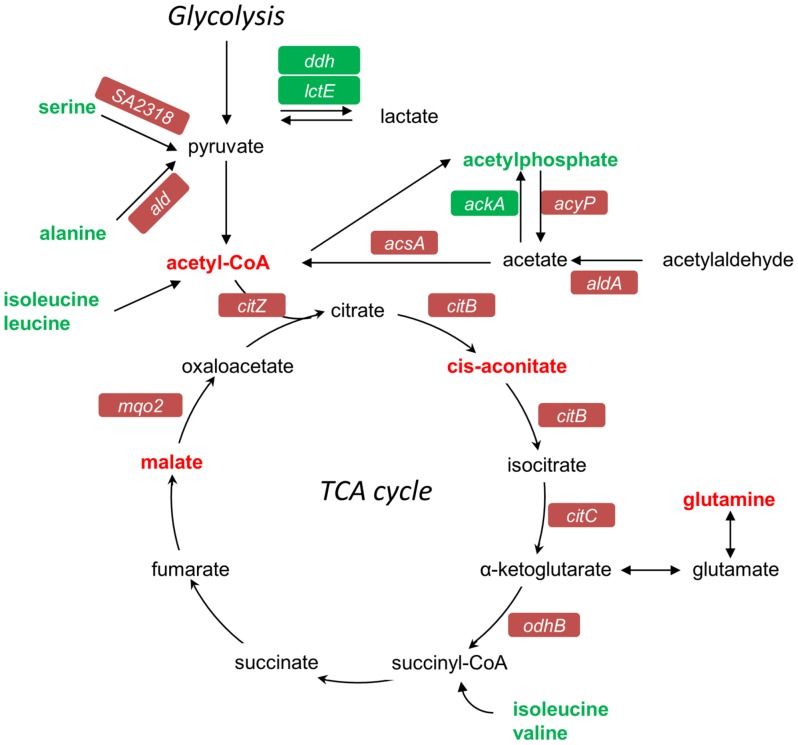
Schematic representation of genes and/or metabolites related to the TCA cycle summarizing changes in both mRNA expression and biochemical levels. Red coloration represents up-regulation while green coloration represents down-regulation during β-lactam mediated HeR/HoR selection.

**Figure 7:**
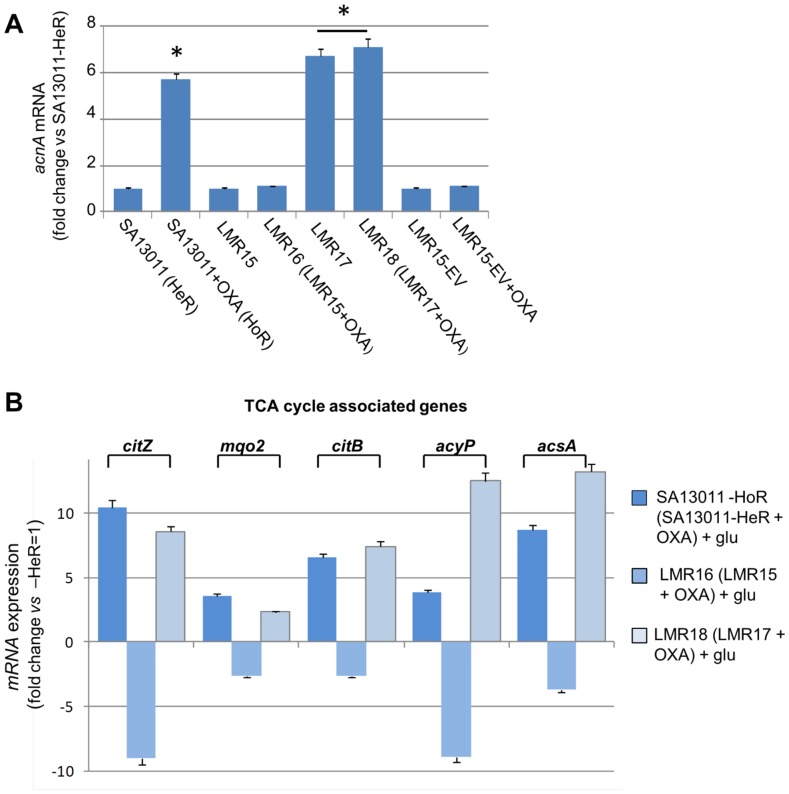
Quantitation of *acnA* mRNA (A) and TCA cycle-associated genes (B) by Real-Time RT-PCR. RNAs were prepared from SA13011-HeR/HoR, *acnA*-null mutant LMR15 and LMR15 complemented with either the empty-vector (LMR15-EV) or wild-type *acnA* (LMR17), grown in the absence or presence of OXA (0.5 µg/ml). Cells were collected at exponential phase of growth as described in Materials and Methods. Relative fold change values *versus* SA13011-HeR ( = 1) of specific mRNAs are shown in the vertical axis; 16rRNA was used as an internal control. *, significantly different than SA13011-HeR (*P<*0.001).h.

**Figure 8:**
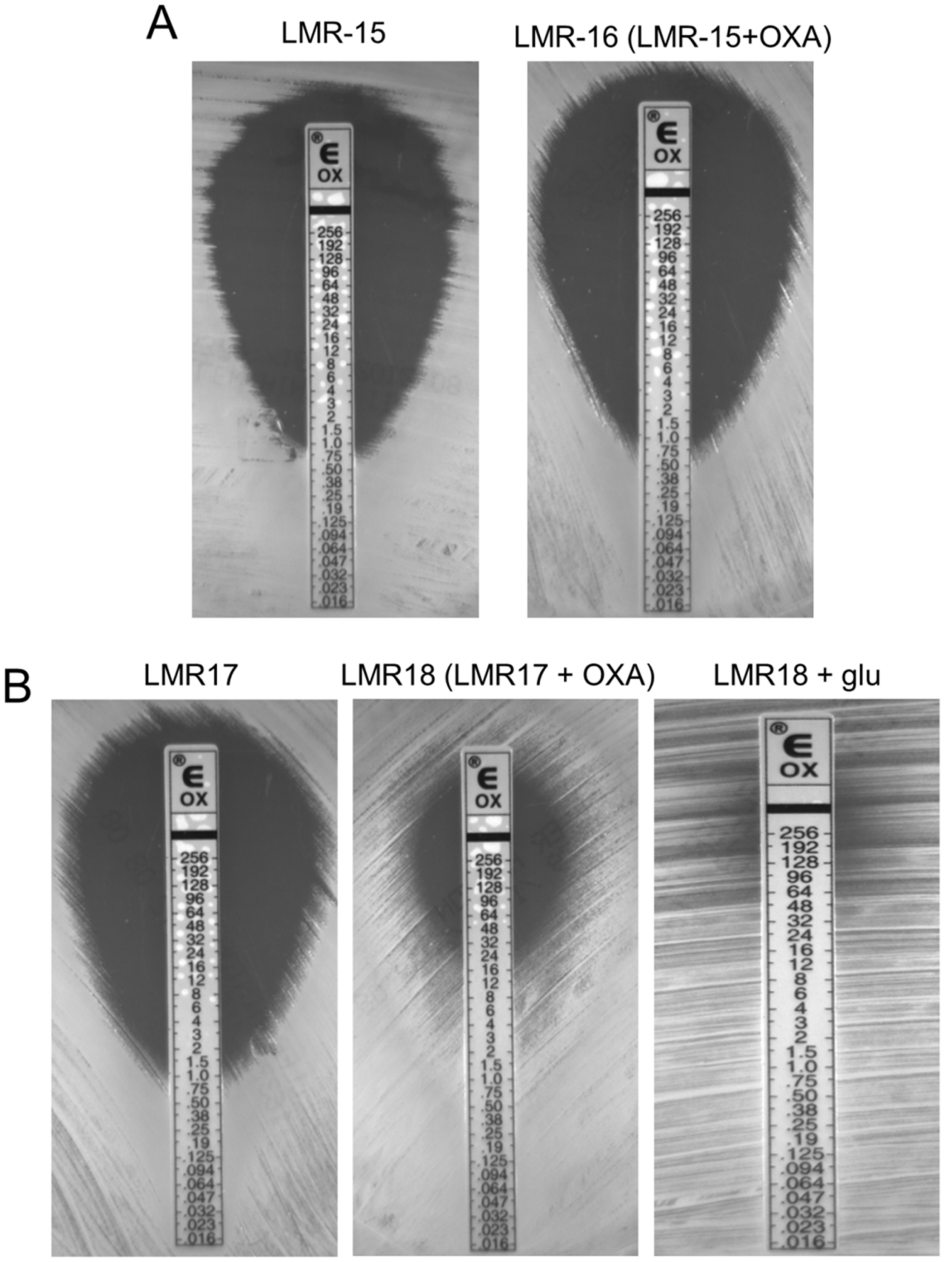
Susceptibility testing of SA13011 aconitase mutant LMR15 and derivatives (−/+ OXA 05 µg/ml; Table 4). Overnight inoculum was diluted to 0.5 Mc Farland standard and swabbed onto MH agar. glu: cells were grown in media supplemented with glucose 10mM. E-test strips were placed on the plates and incubated at 37°C for 24 h. A representative picture of repeated (3) experiments is shown.

The results from gene expression and metabolomics analyses described above demonstrated both a marked increase in the expression of genes corresponding to the TCA cycle and a redirection of metabolic activity toward the cycle (see summary Fig. 6). To further investigate its functional role and the contribution to the β-lactam mediated HeR/HoR selection, the aconitase gene *acnA*-*citB*, which encodes the second enzyme of TCA cycle, was inactivated in SA13011-HeR strain (SA13011 Δ*acnA*::*tetM*, LMR15; Table 4). Expression of *acnA* in mutant strains was monitored by Real-Time RT-PCR (Fig. 7A). Phenotypic analysis of *acnA*-null mutant LMR15 showed no changes in the susceptibility to OXA after exposure to sub-inhibitory concentrations of the antibiotic, i.e. MIC: 1 µg/ml before selection *vs*. 0.75 µg/ml after OXA exposure (Table 3, Fig. 8A). These results indicated that inactivation of the *acnA* gene impaired β-lactam-mediated HeR/HoR selection. Complementation of *acnA*-null mutant LMR15 with a cloned full-length *acnA* (LMR17) resulted in transcription levels similar to those corresponding to SA13011-HoR (Fig. 7A). As a control, LMR15 was complemented with the corresponding empty-vector (LMR15-EV) and, as expected, this did not rescue *acnA* expression (Fig. 7A). Importantly, complementation with full-length *acnA* restored the selection of the OXA resistant derivative, although not to the same degree observed in SA13011-HoR strain [MICs: 32 µg/ml for LMR18 (LMR17+ OXA) *vs.* 256 µg/ml, for SA13011-HoR, Table 3, Fig. 8B). However, higher MICs values were achieved when full-length *acnA*-complemented LMR17 strain undergoing selection with OXA was simultaneously supplemented with glucose (10mM) to maximize optimal glycolysis coverage, which resulted in LMR18 (LMR17+ OXA) MIC of 256 µg/ml (Fig. 8B). Similar results were obtained when pyruvate (20mM) or ribose (12mM) were used instead of glucose (data not shown). Supplementation with carbon sources had no effect *per se* in terms of resistance acquisition either in SA13011-HeR or LMR17 (Table 3). Expression analysis by Real-Time RT-PCR demonstrated altered regulation of TCA cycle genes in the absence of *acnA* during β-lactam-mediated HeR/HoR selection (Fig. 7B). After *acnA* inactivation, primary TCA cycle-related genes including *citZ*, *citB, acyP* and *acsA* were down-regulated in LMR16 (LMR15+ OXA), while complementation of the *acnA* mutant with the cloned full-length gene determined a regain in their corresponding expression in LMR18 (LMR17+ OXA). Together, these results demonstrate the requirement of an active TCA cycle and its key functional role during during β-lactam-mediated HeR/HoR selection process.

**Table 3:** MICs to oxacillin (OXA) corresponding to *S. aureus* aconitase mutants.

STRAIN	MIC OXA (µg/ml)
SA13011-HeR	2
SA13011-HoR (SA13011-HeR+OXA 0.5 µg/ml); SA13011 homogeneous derivative	**256**
LMR15 (SA13011 ΔacnA::tetM)	1
LMR-16 (LMR-15+ OXA 0.5 µg/ml); LMR-15 homogeneous derivative	0.75
LMR17 (LMR15+ wild type acnA)	1
LMR18 (LMR17+ OXA 0.5 µg/ml); LMR-17 homogeneous derivative	**32**
LMR15+ EV	0.5
LMR15+ EV+OXA (0.5 µg/ml)	1
SA13011-HeR+glu	1
SA13011-HeR+OXA (0.5 µg/ml)+glu	**256**
LMR17+ OXA (0.5 µg/ml)+glu	**256**
LMR15+ EV+OXA (0.5 µg/ml)+glu	1

**Table 4:** Strains and plasmids used in this study.

Strain	Relevant Genotype and phenotype	Reference or Source
SA13011-HeR	Heterogeneous [*mecA* (+), OXA susceptible; ST5, SCC*mec* type II, spaType 2, TJMBMDMGMK]	[4], [5]
SA13011-HoR	SA13011-HeR+OXA (0.5****µg/ml); SA13011 homogeneous derivative	[5]
LMR15	SA13011-HeR Δ*acnA*::*tetM*	This study
LMR16	LMR-15+ OXA (0.5 µg/ml); LMR-15 homogeneous derivative	This study
LMR17	LMR15+ wild type *acnA* cloned into pSK265	This study
LMR18	LMR-17+ OXA (0.5 µg/ml); LMR-17 homogeneous derivative	This study
RN4220	Restriction deficient Mutagenized RN450	[5]
SA564 Δ*acnA*::*tetM*	*acnA* mutant	[31]
**Plasmids**		
*E.coli* (PCR2.1-TOPO)	Amp^r^ Kan^r^	Invitrogen
*S. aureus* pSK265	High-copy staphylococcal replicon	[40]

+OXA: indicates the corresponding resistant derivative strain was obtained by growing in the presence of the indicated sub-inhibitory concentrations of OXA.

## Discussion

*S. aureus* is a facultative anaerobe that can survive in aerobic environment during transmission on the skin and with reduced levels of oxygen (anaerobic) during abscess [25]. These observations exemplify the capacity of *S. aureus* to modulate its metabolism accordingly to the encountered conditions. Under these circumstances, *S. aureus* preferentially degrades glucose to pyruvate by the way of the pentose phosphate and glycolic pathways [9]. The catabolic fate of pyruvate is determined by growth conditions; under anaerobic growth, pyruvate is reduced to lactic acid [10] while it is oxidized to acetate and CO_2_ under aerobic conditions [26]. Acetate in the form of acetyl-CoA can be oxidized by the TCA cycle when *S. aureus* is grown in the presence of certain intermediates [27]. In the present study, we were interested in investigating the adaptation of metabolic pathways occurring in the presence of β-lactam during transition from HeR to HoR resistant phenotype by using both global microarrays and metabolomic analyses.

In staphylococci, entry into the post-exponential growth phase usually coincides with the catabolism of non-preferred carbon sources and induction of the TCA cycle [8], which led us to hypothesize that increased resistance to β-lactams (HoR phenotype) alters intermediary metabolism. From this study, we demonstrate that β-lactam-mediated HeR/HoR selection is associated with increased expression of genes that generate acetate, suggesting that acetate generation is one of the main sources supplying the TCA cycle activity. In agreement with this observation, genes encoding for lactate dehydrogenase and alcohol dehydrogenase, the two enzymes that are central to anaerobic fermentation, were found down-regulated. Our results provide strong evidence of the key role played by the TCA cycle during the HeR/HoR selection. In fact, viable inactivation of the cycle through knock-down of the aconitase gene (*acnA*), the second enzyme of the TCA cycle, abolished the capacity of SA13011-HeR to become highly resistant in the presence of β-lactam antibiotics. Importantly, when complemented cells undergoing selection with OXA were supplemented with carbon source (e.g., glucose or pyruvate), resistance phenotypic levels were comparable to the parental HoR resistant strain, demonstrating that active TCA cycle and fueling of it with metabolites entering upper glycolysis steps favored the HeR to HoR selection in MRSA strains. This demonstrates the importance, in addition to OXA-mediated *mecA* increased expression [5], of carbon sourcès ability to be actively metabolized and to allow survival of *S. aureus* HoR cells in the presence of β-lactam antibiotics. Moreover, it is plausible that increased changes in the expression of TCA cycle genes may represent part of a stress response triggered in response to β-lactams. In fact, as we showed in this study, impairment of the TCA cycle and the potential capability of the cell to adapt and redirect its metabolism (aconitase mutant), dramatically altered OXA-mediated HeR/HoR selection, providing strong functional evidence of its involvement and role during the β-lactam-mediated HeR/HoR selection.

In VISA (Vancomycin Intermediate *S. aureus*) strains it has been shown that acetyl-CoA is required for the synthesis of N-acetyl glucosamine and N-acetyl muramic acids, important constituents of murein monomer precursor of cell wall synthesis [8]. Consistent with these observations, we found increased expression of cell wall genes and cell wall precursors during HeR/HoR selection, notably elevated expression of *ald* (alanine dehydrogenase) as well as the three genes involved in glycine degradation (pentaglycine bridges of peptidoglycan) were observed, indicating an increased demand for cell wall biosynthetic components. Numerous examples of the critical role of the TCA cycle in *S. aureus* have been previously reported, showing the significant role played for example, in evasion of immune response [28]. Inactivation of the TCA cycle was shown to delay the resolution of cutaneous ulcers in a mouse soft tissue infection model [28]. Using an *in-vitro* model of aconitase mutant these studies revealed changes in the production of nitric oxide (NO), suggesting that *S. aureus* may enhance its ability to survive in the host by altering its metabolism [28]. Similarly, it has been shown that *S. aureus,* which requires iron to successfully colonize the host [29], is able to redirect its central metabolism to increase iron availability. In a model of iron-starved *S. aureus,* Fur protein-mediated increase in the production of lactate as a fermentative end-product resulted from the concomitant inactivation of TCA cycle enzymes including aconitase [29]. The resulting process, i.e. increased lactate levels, contributed to decrease pH which in turn facilitates the release of iron from host transferrin [29].

Capsule polysaccharide biosynthesis requires TCA cycle intermediates [25]. Inactivation of genes such as *citZ* (citrate synthase), *citC* (isocitrate dehydrogenase) and *citB* (aconitate hydratase) prevents capsule formation, without impairing glucose catabolism but completely inhibited the catabolism of acetate, highlighting the importance of the energy production in the production of virulence factors as well [25]. In line with these observations, we have also found decreased expression of capsule genes in an aconitase null-mutant generated in our laboratory (Singh, C. and Rosato, AE; unpublished observations), which further emphasizes the key role that both the TCA cycle and the re-direction of other metabolic pathways may have in providing cells the capacity to develop the high resistant phenotype.

In summary, the present study highlights the importance of metabolic adaptations of heterogeneous MRSA clinical strains when undergoing selection to highly resistant HoR derivatives in the presence of β-lactam antibiotics. These results postulate that β-lactam-mediated HeR/HoR selection is associated with severe metabolic stress, as demonstrated by increased production of acetyl-CoA, increased catabolism of fatty acids (β-oxidation) and amino acids, and decreased oxidative phosphorylation, altogether contributing to increased TCA activity that supports and promotes survival in the presence of β-lactam antibiotics. Importantly, these observations may identify a promising avenue for combating multidrug-resistant bacteria, as recently observed with compounds that were directed against the *S aureus* pyruvate dehydrogenase complex [30].

## Materials and Methods

### Bacterial Strains

Clinical MRSA strain SA13011 and derivatives are shown in Table 4. SA13011 is representative of a heterogeneous MRSA collection previously described [4], [5], which were determined as OXA susceptible and *mecA* positive [4], [5]. For this study, isogenic heterogeneous *S. aureus* 13011 strain (HeR; OXA MIC: 2 µg/ml) and its highly homogeneous methicillin resistant derivative, (SA13011-HoR; OXA MIC: 256 µg/ml) were used. SA13011 was part of a group of 25 isolates first described in a previous study which was identified as ST5, SCC*mec* type II, spaType 2, TJMBMDMGMK (13 of the 25 isolates including SA13011 presented these characteristics) [4].

### Antibiotics and Chemicals

All the antibiotics and chemicals used in this study including oxacillin OXA (used at concentrations of 0.5 µg/ml), chloramphenicol (10 µg/ml), tetracycline (5 µg/ml); and carbon sources pyruvate (10 mM), glucose (20 mM) and ribose (12 mM) were purchased from Sigma-Aldrich (St. Louis, MO) and Thermo-Fisher Scientific (Waltham, MA).

### Growth Conditions

Selection of SA13011 from the heterotypic (HeR) to the homotypic (HoR) resistance phenotype was performed as we previously described [5]. Briefly, bacteria were grown overnight in 5 ml LB broth without antibiotic, diluted to an optical density at 600 nm (OD_600_) of ∼0.025 in 300 ml LB broth, either with or without 0.5 µg/ml OXA, and grown at 37°C with shaking (180 rpm). The ODs were monitored every hour for up to 35 h. β-lactam-mediated HeR to HoR selection was verified by streaking the cells onto an OXA gradient plate with a concentration ranging from 0 to 128 µg/ml, as previously shown [5]. SA13011-HoR resistant cells were proven stable after several passages in free-antibiotic media as previously shown [3], [5].

### Mutational Insertion Inactivation of Aconitase (*acnA-citB)* and Complementation

The *acnA*-null mutant was constructed by moving *acnA*::*tetM* from strain UAMS-1[31] into SA13011-HeR by general transduction using 80α phage [32]. Trans-complementation of *acnA* was performed by using a construct encompassing the complete *acnA* gene as well as the upstream region (0.425 kb) including the putative ribosomal binding site and promoter using *acnA* primers F and R shown in Table S2. The 3.7 kb PCR fragment product was purified using the QIAquick gel extraction kit (Qiagem, Valencia, CA), ligated into the ligase-independent cloning site of the PCR2.1-TOPO vector (Invitrogen, Life Technologies, Carlsbad, CA), and transformed into chemically competent TOP10 *E. coli* (Invitrogen). A staphylococcal origin of replication was introduced by cloning plasmid pSK265, *S. aureus* replicon [14] into the unique *BamHI* site on PCR 2.1-TOPO (Table 4); the construct was moved into *S. aureus* RN4220 by electroporation [3]. Trans-complementation of *acnA* mutant was obtained by transduction of plasmid psk265 containing wild-type *acnA* from RN4220 by phage 80α into SA13011 *acnA* null mutant (SA13011*-ΔacnA::tet*).

### Samples Extraction and Metabolic Profiling

The metabolomic analysis was performed by Metabolon, Inc. (Durham, NC). The untargeted metabolic profiling platform employed for this analysis combined three independent platforms: ultrahigh performance liquid chromatography/tandem mass spectrometry (UHLC/MS/MS^2^) optimized for basic species, UHLC/MS/MS^2^ optimized for acidic species, and gas chromatography/mass spectrometry (GC/MS). Samples were processed essentially as described previously [33]–[38]. Five biological samples served as replicates throughout the data set; extracted water samples served as process blanks, and a cocktail of standards spiked into every analyzed sample allowed instrument performance monitoring. In addition, three types of controls were analyzed simultaneously with the experimental samples.

### Metabolite Identification and Data Analysis

Metabolites were identified by automated comparison of the ion features in the experimental samples to a reference library of chemical standard entries that included retention time, molecular weight (*m/z*), preferred adducts, and in-source fragments as well as associated MS spectra and curated by visual inspection for quality control using software developed at Metabolon [37].

Experimental samples and controls were randomized across a one-day platform run. Any missing values were assumed to be below the limits of detection and for statistical analyses and data display purposes, these values were imputed with the compound minimum (minimum value imputation) after normalization to total protein measurement (Bradford) for each sample. Following log transformation of protein normalized imputed values, Welch’s two-sample *t*-tests were used to identify biochemicals that differed significantly (*p*≤0.05) between experimental groups using Array Studio software (OmicSoft) (Table S1). Multiple comparisons were accounted for by estimating the false discovery rate (FDR) using q-values [37] (Table S1). Hierarchical clustering was performed with Array Studio software (OmicSoft) using complete linkage method and correlation distance metric.

### Analysis of Gene Expression by Microarray Transcriptional Profiling and Real-Time RT-PCR

RNA extractions for real-time RT-PCR were performed as previously described [5], [13]. Total RNA was extracted using a RNeasy isolation Kit (Qiagen); all RNA samples were analyzed by *A*_260_/*A*_280_ spectrophotometry and gel electrophoresis to assess concentration and integrity, and cleaned of potential DNA contamination by treating them with DNAse as per manufacturer recommendations (Ambion, Life Technologies, Austin, TX). Pair-wise comparisons were made in biological triplicates between representative strains and collected at similar exponential growth phase [14]. Microarray transcriptional profiles were carried out as previously described [5], [14] by using a spotted DNA microarray (TIGR version 6 *S.aureus* slides), containing 4546 oligos (70mer) covering the genomes of *S.aureus* COL (2654 ORFs), N315 (2623 ORFs), Mu50 (2748 ORFs), MRSA 252 (2744 ORFs), MSSA 476 (2619 ORFs) and pLW043 (62 ORFs), as previously described [5], [13]. TIFF images of the hybridized arrays were analyzed using TIGR-Spotfinder software (http://www.tigr.org/software/). The data set was normalized by applying the LOWESS algorithm (block mode; smooth parameter: 0.33) and using TIGR-MIDAS (http://www.tigr.org/software/) software, and significant changes were identified with SAM (significance analysis of microarrays; http://www-stat.stanford.edu/~tibs/SAM/index.html) software. Differential expression was defined as a change of more than two-fold in transcript versus the comparator strain.

Real-time reverse transcription-PCR analysis was done using the SensiMix SYBR One-Step kit (Quantace/Bioline, Taunton, MA) according to the manufacturer's protocol. Gene expression was compared according to the *C*_T_ values converted to fold change with respect of a sample considered as reference (value = 1) using log_2_–(ΔΔCt). The change (*n*-fold) in the transcript level was calculated using the following equations: Δ*C_T_* = *C_T(test DNA)_* − *C_T(reference cDNA)_*, ΔΔ*C_T_* = Δ*C_T(target gene)_* − Δ*C_T(16S rRNA)_*, and ratio = 2^−ΔΔC^ *_T_* [39]. The quantity of cDNA for each experimental gene was normalized to the quantity of 16S cDNA in each sample as determined in a separate reaction. Each RNA sample was run in triplicate. Values represent the means of at least three biological replicates ± standard error of the mean (SEM), sampled in triplicate to minimize error by inter- and intra-samples. Differences between the mean values were analyzed using a one-way analysis of variance (ANOVA). A *P* value of <0.01 was considered statistically significant (*). Oligonucleotide primers are shown in Table S1.

## Supporting Information

Figure S1 **Analysis of biochemicals corresponding to amino-acid metabolism during β-lactam mediated HeR/HoR selection.**
(TIF)

Figure S2 **Analysis of biochemicals corresponding to glutathione metabolism during β-lactam mediated HeR/HoR selection.**
(TIF)

Figure S3 **Quantitation of mRNA levels of TCA cycle-, carbohydrate catabolism- and cell wall-associated genes by Real-Time RT-PCR during SA43002 (phenotypically similar to SA13011) β-lactam induced HeR/HoR selection.** RNA was prepared from SA43002-HeR and its highly resistant derivative SA43002-HoR (SA43002-HeR+OXA 0.5 µg/ml) cells, collected at exponential phase of growth, as described in Materials and Methods. Relative fold change values of specific mRNAs in SA43002-HoR vs. SA43002-HeR (reference value = 1) are shown on the vertical axis. Relative fold change values representing the means of at least three biological replicates of specific mRNAs ± standard error of the mean (SEM), sampled in triplicate to minimize error by inter- and intra-samples, are shown on the vertical axis; 16S rRNA was used as an internal control. Oligonucleotide primers are shown in Table S1.
(TIF)

Table S1 **Heat map of statistically significant biochemicals profiled in this study.** Shaded cells indicate p≤0.05 (red indicates that the mean values are significantly higher for that comparison; green values significantly lower). Blue-bolded text indicates 0.05<p<0.10. All data are normalized to Bradford protein assay values.
(XLSX)

Table S2 **Primers used in this study.**
(DOCX)
