# Supplementary figures and images for: Exposure of Clinical MRSA Heterogeneous Strains to β-Lactams Redirects Metabolism to Optimize Energy Production through the TCA Cycle
Source: PLoS One. 2013 Aug 5;8(8):e71025. doi: 10.1371/journal.pone.0071025 (PMC3733780; doi:10.1371/journal.pone.0071025)

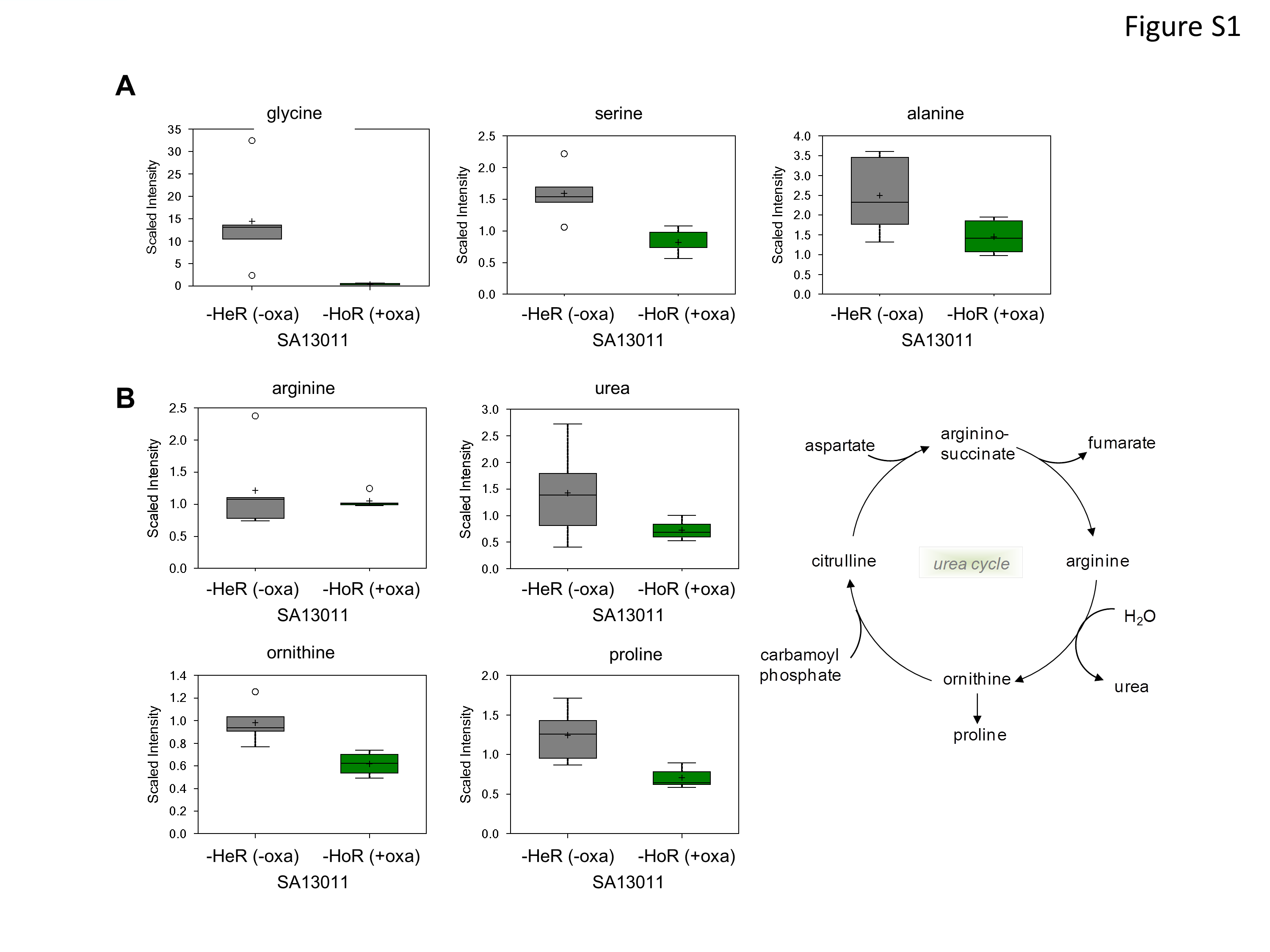

Supplement: Figure S1 — Analysis of biochemicals corresponding to amino-acid metabolism during β-lactam mediated HeR/HoR selection. (TIF) [file pone.0071025.s001.tif]

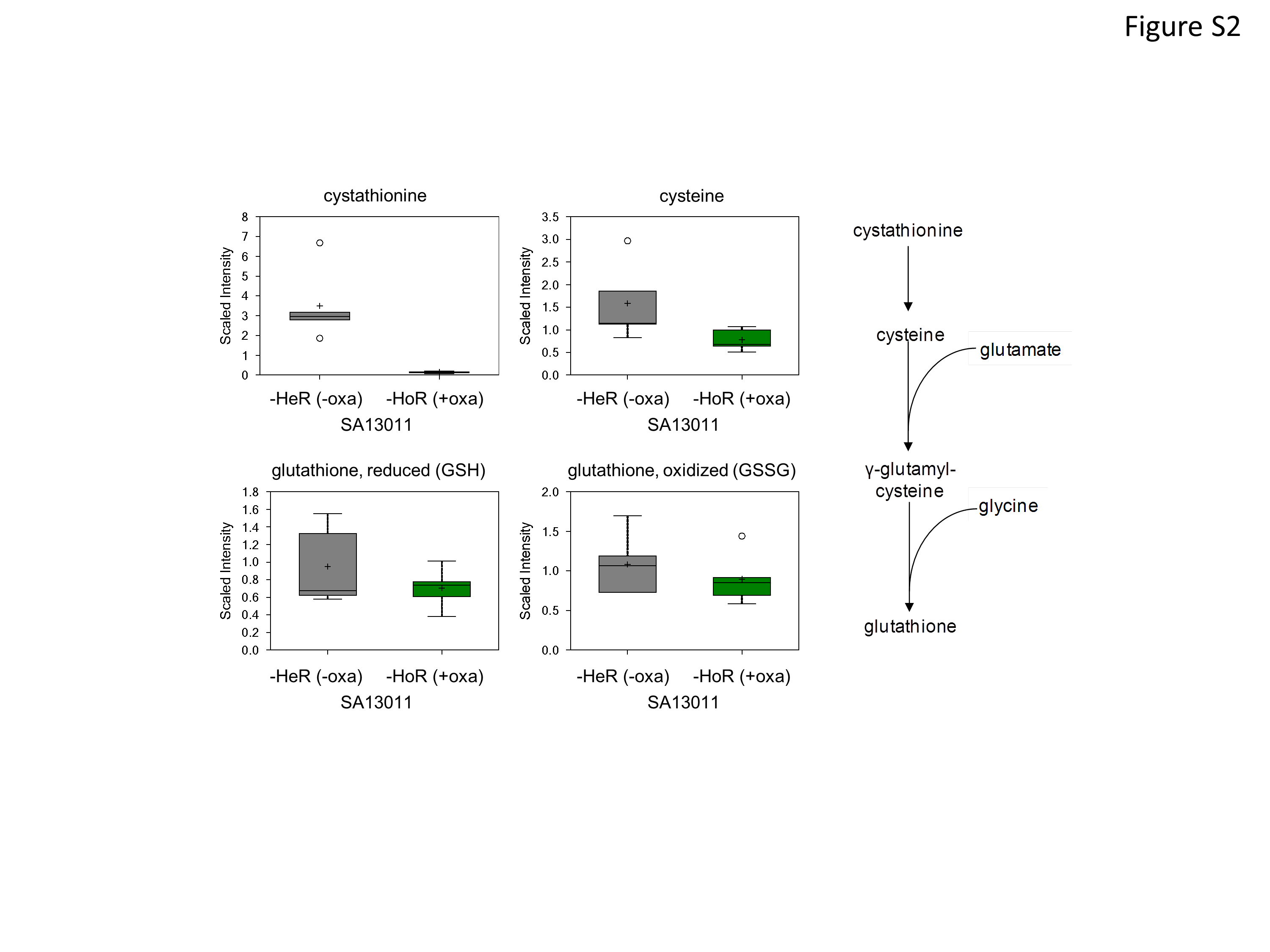

Supplement: Figure S2 — Analysis of biochemicals corresponding to glutathione metabolism during β-lactam mediated HeR/HoR selection. (TIF) [file pone.0071025.s002.tif]
