# Supplementary material for: Exposure of Clinical MRSA Heterogeneous Strains to β-Lactams Redirects Metabolism to Optimize Energy Production through the TCA Cycle
Source: PLoS One. 2013 Aug 5;8(8):e71025. doi: 10.1371/journal.pone.0071025 (PMC3733780; doi:10.1371/journal.pone.0071025)
Supplement: Table S2 — Primers used in this study. (DOCX) [file pone.0071025.s005.docx]

**Table S2.** Primers used in this study.

| **Primer Name** | **Sequence** |
| --- | --- |
| *citB*-F | GGAAGAATGATGGATTTTGAATGTATG |
| *citB*-R | GAAGTTTCAGATACCGTTGATAGTG |
| *citZ*-F | GGGTCAATTGAAAATGTTGATGC |
| *citZ-*R | CAAATAATTCTTCACGACCAGCGTC |
| *mqo2-*F | GATTTATTCAAGTCTGTTAAAACATACAAC |
| *mqo2-R* | GTGTATAATTGCCAATCTTCATTACGTGC |
| *ackA*-F | GGATTCAATTTTTACAATTGAAGTCAACGGGG |
| *ackA*-R | CATCAGTAATAGCTACTGATTCAGGG |
| *acyP-F* | GCAATGAACTATAACATTGTCGGTACTG |
| *acyP-R* | CATCAGTAATAGCTACTGATTCAGGG |
| *ald-F* | ATGGCAGTAAACGTTCGAG |
| *ald-R* | CTGCATGATCGACATCTTTA |
| *gcvt-F* | GTGGTATCGCTTTTGCAAGTAAAC |
| *gcvt-R* | CTTCATAACCAGTTCTTGCAATTC |
| *SA2318-F* | GCCGTAGAAAATAATAGTCA |
| *SA2318-R* | GCCATCACCTGTCGTCCCTT |
| *rocA-F* | GGAACTTCCCGTTTGCGATTATGG |
| *rocA-R* | CACCAATTTCTTTCGGATCACCAGG |
| *rocD-F* | GTGCTGCATCAATTGCTGCATTAGATG |
| *rocD-R* | CAAAGCTTCACAATATGGTCTAGC |
| *dltA-F* | CTTAGATCAATATCCGACATTACCTG |
| *dltA-R* | CGAATACCGTCATCGAAATTAAATAC |
| *dltC-F* | GCAGAAGTAGCAGAAAATGATATTG |
| *dltC-R* | CATCGTAACTCTTCTAATGCTTCAAC |
| *rbsD-F* | CAAAAGCAATCGCGACAATTGGTC |
| *rbsD-R* | GGGTTATGTTCTTTTATTTCTTCTGC |
| *rbsK-F* | AGTAGCAAAATATGTACCTTGCTTACCTAG |
| *rbsK-R* | CGCTATAATCAATGCGACTTTGTCGTT |
| *mecA-F* | GGTGAAGTAGAAATGACTGAACGT |
| *mecA-R* | AATGACGCTATGATCCCAATCTAAC |
| *pbp2-F* | TATTTAGCCGGTTTACCTCA |
| *pbp2-R* | TTTTGACGTTCTTCAGGAGT |
| *femA-F* | GTTAGTACCTTTAGCGTATATCAA |
| *glmS-F* | GTAAGTTTAGAAGGTGCGTTAAAC |
| *glmS-R* | CATGGATGTGCACCACGTGCTACTAC |
| *glpK-F* | GGGACGATGAGTTATTAGAACTAC |
| *glpK-R* | GTGACGTGAAAAACACATATGGAAC |
| *femA-F* | GTTAGTACCTTTAGCGTATATCAA |
| *femA-R* | GTAAAGTTGGACCCAAAAGAA |
| *tagF-F* | ATGAAACGTGTAATAACATATCGA |
| *tagF-R* | CAAGCATCATTTTTCGTTGTTCA |
| *glmS-F* | GTAAGTTTAGAAGGTGCGTTAAAC |
| *glmS-R* | CATGGATGTGCACCACGTGCTACTAC |
| *glpK-F* | GGGACGATGAGTTATTAGAACTAC |
| *glpK-R* | GTGACGTGAAAAACACATATGGAAC |
